# ModFOLD8: accurate global and local quality estimates for 3D protein models

**DOI:** 10.1093/nar/gkab321

**Published:** 2021-05-08

**Authors:** Liam J McGuffin, Fahd M F Aldowsari, Shuaa M A Alharbi, Recep Adiyaman

**Affiliations:** School of Biological Sciences, University of Reading, Whiteknights, Reading RG6 6AS, UK; School of Biological Sciences, University of Reading, Whiteknights, Reading RG6 6AS, UK; School of Biological Sciences, University of Reading, Whiteknights, Reading RG6 6AS, UK; School of Biological Sciences, University of Reading, Whiteknights, Reading RG6 6AS, UK

## Abstract

Methods for estimating the quality of 3D models of proteins are vital tools for driving the acceptance and utility of predicted tertiary structures by the wider bioscience community. Here we describe the significant major updates to ModFOLD, which has maintained its position as a leading server for the prediction of global and local quality of 3D protein models, over the past decade (>20 000 unique external users). ModFOLD8 is the latest version of the server, which combines the strengths of multiple pure-single and quasi-single model methods. Improvements have been made to the web server interface and there has been successive increases in prediction accuracy, which were achieved through integration of newly developed scoring methods and advanced deep learning-based residue contact predictions. Each version of the ModFOLD server has been independently blind tested in the biennial CASP experiments, as well as being continuously evaluated via the CAMEO project. In CASP13 and CASP14, the ModFOLD7 and ModFOLD8 variants ranked among the top 10 quality estimation methods according to almost every official analysis. Prior to CASP14, ModFOLD8 was also applied for the evaluation of SARS-CoV-2 protein models as part of CASP Commons 2020 initiative. The ModFOLD8 server is freely available at: https://www.reading.ac.uk/bioinf/ModFOLD/.

## Graphical Abstract

**Graphical Abstract:**
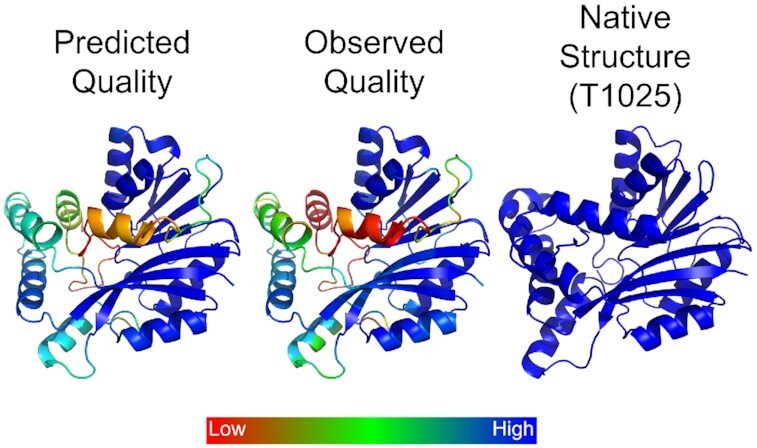
ModFOLD8 local quality estimates for a predicted 3D protein model.

## INTRODUCTION

The prediction of protein tertiary structures has become a routine part of molecular biology and there is a plethora of servers for building 3D atomic models from amino acid sequences. Each server may use different algorithms, databases and sets of templates in order to construct models, or they may carry out template free modelling using various techniques, for example based on fragment assembly and/or deep learning. Some modelling algorithms may be better than others given different situations, but any one pipeline may generate dozens of alternative 3D models for a given sequence. Therefore, if many servers are queried, then potentially hundreds of different alternative models may be available to researchers for the same protein target. However, most researchers are only interested in identifying the most accurate models – those that are most likely to be closest to the native structures. Furthermore, they need to have confidence in the accuracy of the predictions as well as knowledge about how close models are likely to be to the native structures. To gain such information, model Quality Assessment (QA) methods, such as the ModFOLD servers (1–3), must be used. QA methods can help to answer the three major questions facing researchers predicting 3D models of proteins. Firstly, which 3D protein models are the best? Secondly, how good are the models? Thirdly, where are the errors in the models located?

In the Critical Assessment of Structure Prediction (CASP) experiments, the methods for QA are classified into 2 broad categories. Firstly, the single model methods, which consider only the information within an individual model (recent top methods have included ModFOLD7, ProQ3D, FaeNNz and VoroMQA) (4,5). Secondly, the clustering/consensus approaches, which make structural comparisons between multiple models for the same target (for example, UOSHAN, MULTICOM_CLUSTER & ModFOLDclust2) (4,5). Historically, multiple model methods have been more accurate than single-model methods, but they are more computationally intensive and do not work well when very few or many very similar models are available. Until recently, single model methods have been less accurate overall, but they are more rapid, they produce consistent scores for single models at a time, and they often perform better at model ranking and selection. The ModFOLD server uses a hybrid approach to provide quality estimates for single models, through the integration of several pure-single model scores along with quasi-single model scores, which generate reference sets of models from the target sequence prior to making structural comparisons. The latest version of ModFOLD builds on our previous successes with this hybrid strategy (3,4,6–8), through the integration of additional component methods exploiting advances in deep learning and residue contact predictions. We report on recent successes in recent CASP experiments as well as highlighting the progress of the method's development in the CAMEO benchmarks.

In the latest CASP14 experiment, ModFOLD8 ranked among the top few performing methods for model quality estimates according to the official assessors, and we were invited to speak on the round table along with members from the Yang, Baker and tFOLD-IDT groups. CASP14 was notable in the context of the very high-quality models of tertiary structures produced by some groups for targets with no known templates. While this progress in modelling is a major achievement, it is important to note that most models are imperfect and still contain significant local errors, especially those produced by the structure prediction servers that are currently available to the wider community. ModFOLD8 can successfully detect such errors, including those in the high-quality models that are closer to experimental structures. This information is essential for the successful utility and application of models for further biological investigations, and for their wider acceptance by the bioscience community. As protein 3D modelling methods improve, it is vital that users can build trust in them by using freely available and independent quality assessment methods, such as ModFOLD8.

## MATERIALS AND METHODS

**Figure 1.**
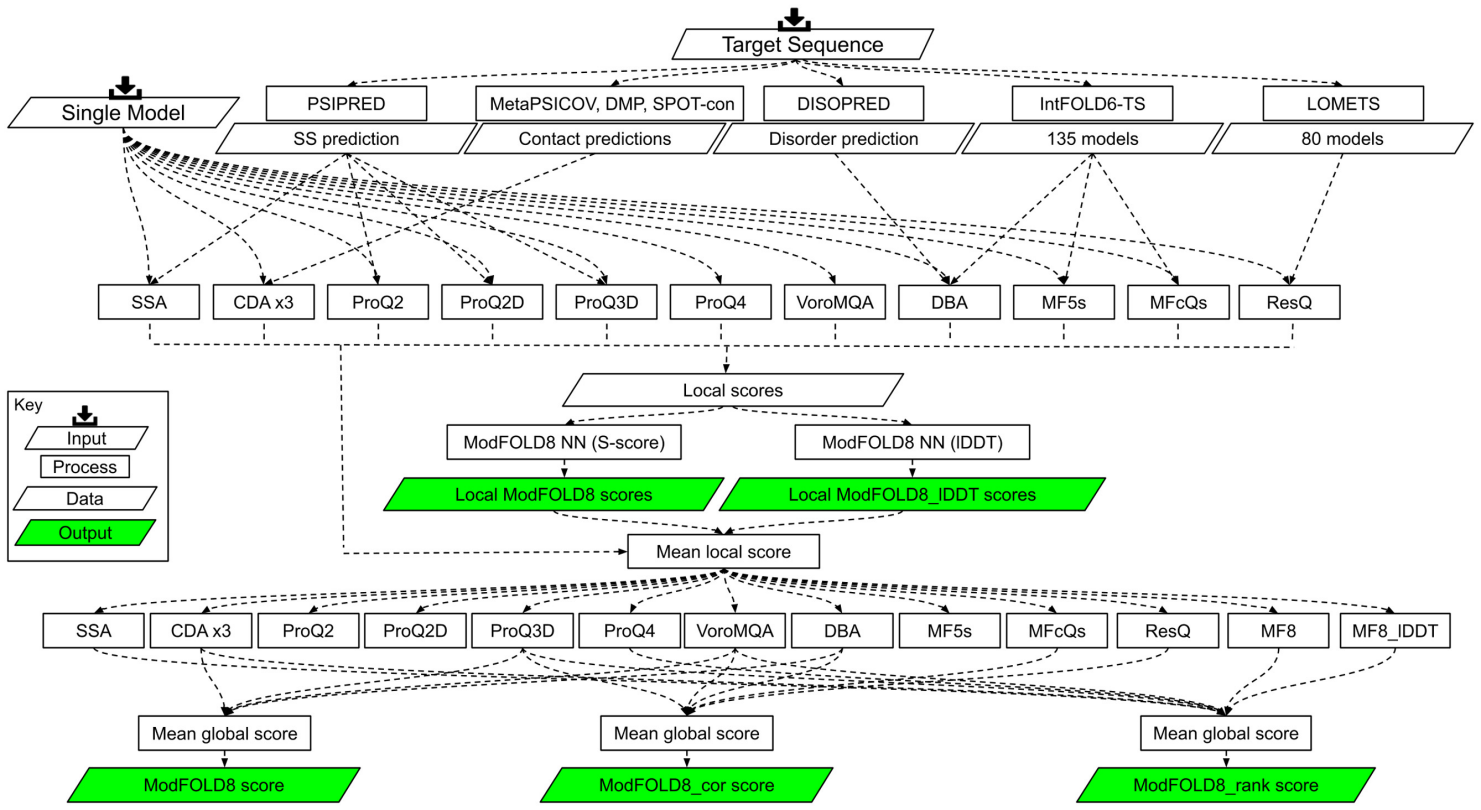
Flow chart showing data and processes for the ModFOLD8 methods. The inputs at the top are simply a single 3D model and the target sequence. The target sequence was then pre-processed to produce predicted secondary structures, contacts, disorder and reference model sets. These data were then fed into the individual local/per-residue scoring methods. Subsequently, these local scores were fed into the two different neural networks, trained to predict the S-scores and lDDT scores. The global scores for each method were calculated from the mean local scores. Different combinations of the global scores were used to generate the final ModFOLD8_rank, ModFOLD8_cor and ModFOLD8 global scores.

Our aim with ModFOLD8 was to increase prediction accuracy by building further on the individual strengths of multiple pure-single and quasi-single model methods. Thus, ModFOLD8 combines inputs from 13 different scoring methods (nine pure single model inputs and four quasi-single model inputs), using neural networks (NNs) (Figure 1).

The nine pure single model inputs included the ProQ methods (ProQ2 (9), ProQ2D (10), ProQ3D (10) and ProQ4 (4)), VoroMQA (11), and four other methods developed in our group, comprising three different Contact Distance Agreement (CDA) scores and the Secondary Structure Agreement score (SSA) (3,6). The CDA_DMP and CDA_SC scores are the two new pure single model scoring methods, based on our original CDA score (3,6), which measures the agreement between the predicted residue contacts according to MetaPSICOV (12) and the measured Euclidean distance (in Å) between residues in the model. However, the contact predictions from DeepMetaPSICOV (13) and SPOT-Contact (14) were used as inputs for CDA_DMP and CDA_SC respectively.

The four quasi single model inputs included ResQ (15) and three other methods developed in our group: Disorder *B*-factor Agreement (DBA), ModFOLDclust_single (MF5s) and ModFOLDclustQ_single (MFcQs) (3,6). These quasi-single model methods all rely on the generation of reference sets of models for comparison. The DBA, MF5s and MFcQs scores all compare the input model versus 135 reference models generated using our latest version of IntFOLD (16), while ResQ uses the reference models from the LOMETS method (17).

For producing final local score outputs, we used a simple multi-layer perceptron (MLP) for each neural network, and we trained them using the input scores from the 13 methods (Supplementary Figure S1). The first NN variant was trained using the *S*-score (3,6) and second variant was trained using the lDDT score (18) as the target functions. The NN inputs consisted of a sliding window (size = 5) of per-residue scores from all 13 of the scoring methods described above, and the output was a single quality score (i.e. either the *S*-score or lDDT) for each residue in the model, giving 65 input neurons, with 33 hidden and 1 output. The RSNNS package for R was used to construct the NNs, which were trained using data derived from the evaluation of CASP11 server models versus native structures. The similarity scores were used for ease of training the NN; the MLP learns more effectively when inputs and outputs are scaled 0–1. For both of the per-residue methods, the similarity scores, s, for each residue were converted back to distances, *d*, i.e. the predicted distances in Å of each Cα atom from the native structure, using the inverse S-score function: *d* = 3.5√((1/*s*) − 1).

For producing global score outputs, we made 3 variants that combined the mean global scores from different methods. Each global score was optimised for the main aspects of the quality estimation problem. Firstly, ModFOLD8_rank, which was optimised for ranking (i.e. the top ranked models should be closer to the highest observed accuracy, but the relationship between predicted and observed scores may not be linear). Secondly, ModFOLD8_cor, optimised for correlations with the observed scores (i.e. the predicted global quality scores produced should produce closer to linear correlations with the observed global quality scores). Finally, ModFOLD8, with more balanced performance both for correlations of predicted and observed scores and rankings of the top models (Figure 1).

## RESULTS AND DISCUSSION

### Server inputs and outputs

The required inputs for ModFOLD8 are the amino acid sequence for the target protein and a single model 3D model for evaluation. Optionally, users may upload multiple alternative models, a name for their protein sequence and their email address. Providing a reference sequence allows all submitted models (including partial models) to be compared fairly using the same calculated sequence-based input data. It is also useful for ensuring consistent residue numbering for all submitted models. The time taken for a prediction will depend on the length of sequence, the number of models submitted and the load on the server. For a new run on a single model, users should typically receive results back within 24 h, once the job is running. Large batches of models (several hundred) for a single target may take several days to process. However, if a model has already been submitted for the same target sequence within the same week, then the reference model library for that sequence will already be available to the server (the results will be cached) and so users should receive their results back much more quickly, typically within a few hours.

**Figure 2.**
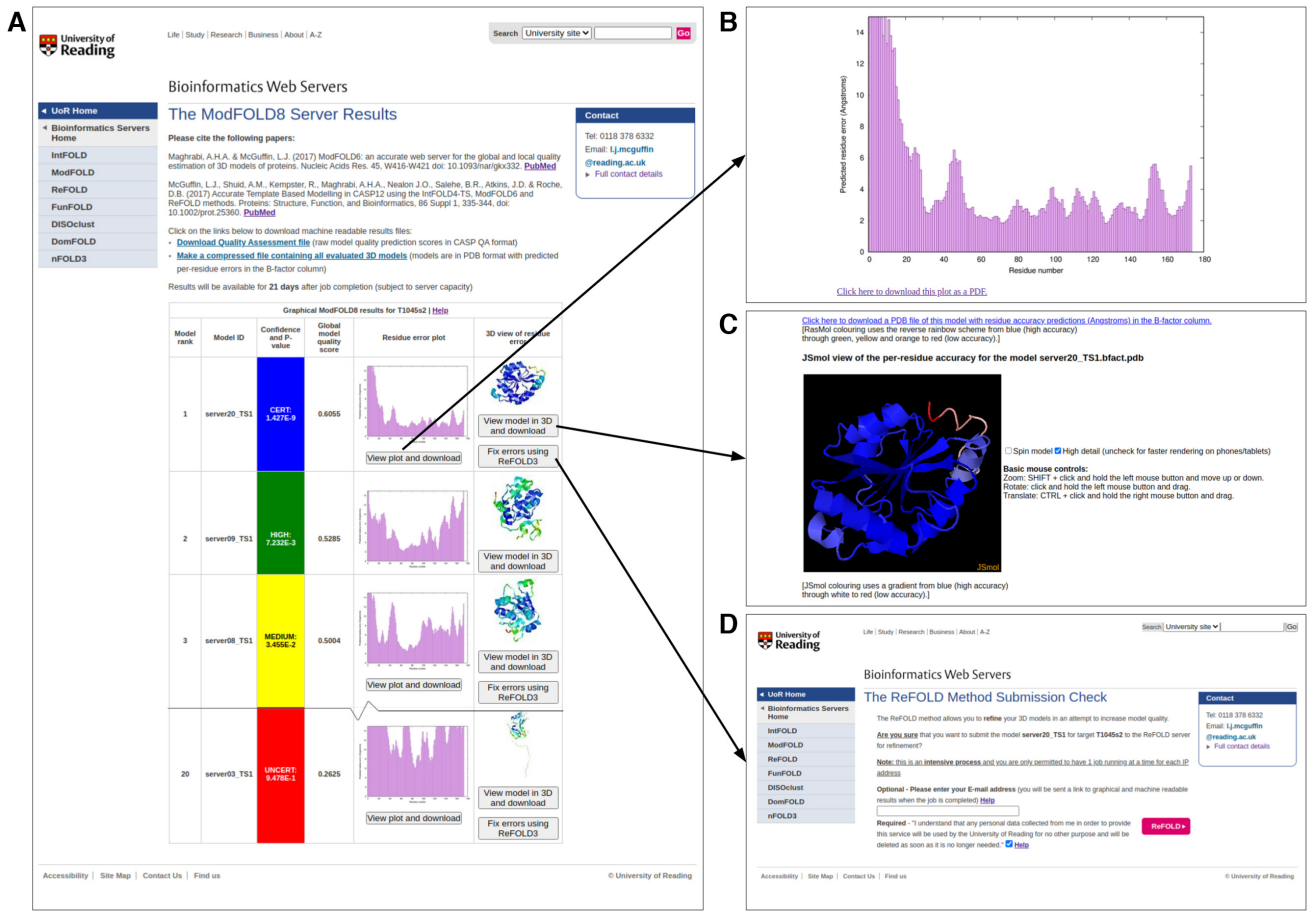
ModFOLD8 server results for the CASP14 target T1045s2. (**A**) Main results page showing summary of graphical output for each model (table is truncated to fit page). The arrows point to additional graphical results that are accessed when users click on the buttons on the main page. (**B**) The per-residue error plot showing the errors for each residue in the model (predicted distance in Å of each Cα atom from the native structure), which can be downloaded as a PDF. (**C**) Interactive JSmol view of the model. Users can also download their models in PDB format with the predicted residue errors shown in the b-factor column. (**D**) The ‘Fix errors using ReFOLD3’ button allows users to submit their 3D models to the ReFOLD server (19) (version 3) for refinement guided by the local quality scores.

Figure 2 shows the graphical interactive results data obtained from the ModFOLD8 web server. Figure 2A, shows a screenshot of the results page which consists of a single table summarising the quality scores for each submitted model with plots of local errors and images of annotated 3D models. Each row in the table includes, the model rank and ID, the global scores, a confidence score and *P*-value, and thumbnails of the graphical results (error plots and images of each model coloured by local quality). The push buttons provided allow users to: view the per-residue error plots for each model and download them as PDFs (Figure 2B), download and view their ‘*b*-factor’ annotated models in 3D interactively within the browser (Figure 2C), and refine their models to fix the identified errors via the latest version of our ReFOLD server (19) (Figure 2D). Finally, users may download the raw machine-readable files, containing the quality estimation data in CASP format, as well as compressed archives for all the annotated models.

### Independent benchmarking and cross validation

#### Cross validation

The results from our in-house benchmarking indicate that the ModFOLD8 methods outperform our previous versions, ModFOLD6 (3,6) and ModFOLD7 (4,7), on the same CASP11 data set. The charts in Supplementary Figure S2 show the progressive incremental increases in accuracy from ModFOLD6 to ModFOLD8. According to the ROC analysis, ModFOLD8 outperforms our previous versions in terms of local score accuracy evaluated by both the S-score and the lDDT score. Furthermore, the ModFOLD8_cor and ModFOLD8_rank variants outperform their equivalent previous variants in terms of global score accuracy (Supplementary Figure S2).

#### CAMEO

The progressive increases in performance between versions of ModFOLD has also been continuously independently evaluated over the years, via the Quality Estimation (QE) category of the CAMEO resource (20,21). The data in Supplementary Figure S3 and Supplementary Tables S1 and S2, show the performance gains for each version of the ModFOLD server, in terms of the local accuracy measured by the lDDT score, compared with the best available public servers from each developer group (ModFOLD8 is currently listed as Server 39). Further to the QE category, the ModFOLD8_rank method is used to evaluate and select models as part of the IntFOLD6 server, which is continuously benchmarked in the 3D category of CAMEO. According to the CAMEO results, IntFOLD6 (Server 90) has shown improved performance over our last three methods IntFOLD3 (22), IntFOLD4 (6) and IntFOLD5 (16) (https://www.cameo3d.org/modeling/server/1-year/id/server90/difficulty/all/subset/?to_date=2021-01-16&server_list=75_3D,58_3D,33_3D).

#### CASP13, CASP14 and CASP Commons 2020

Table 1 shows results from the latest independent blind community wide CASP14 experiment in the QA category, where the predicted global accuracy scores produced by methods are compared in terms of their differences to the observed scores (lower scores indicate higher performance). Alternative official evaluation measures from CASP13 and CASP14 are shown in Supplementary Tables S3–S15. In the last two CASP experiments, which have occurred have since our last paper in this journal describing the ModFOLD6 release (3), ModFOLD7 and ModFOLD8 have ranked within the top 10 performing methods in most official independent evaluations, according to the main GDT_TS or lDDT metrics (Supplementary Tables S3–S15, https://predictioncenter.org/casp14/, https://predictioncenter.org/casp13/). Just prior to CASP14, our group participated in the CASP Commons 2020 community wide effort to model the harder protein targets from SARS-CoV-2. We used the ModFOLD8_rank variant to evaluate the server models, which produces global scores that are optimised for model selection. Supplementary Figure S4 shows the top AlphaFold (23) and AlphaFold2 models, for the two C19 targets, which have structures in the PDB (post CASP14). These results demonstrate that by mapping the ModFOLD8 local scores onto models, the more poorly modelled regions in the predicted structures can be discriminated approximately from regions that are likely to be closer to the native structures.

**Table 1.** Official CASP14 global QA evaluation (Differences in predicted versus observed scores, stage 2 – best 150). Only the top 10 groups are shown (there are 72 groups in total). Table is sorted by the LDDT score. Lower scores indicate higher performance. Data are from https://predictioncenter.org/casp14/qa_diff_mqas.cgi

Rank	Group	Model	GDT_TS	LDDT	CAD (AA)	SG
1	**ModFOLD8_rank**	QA120_2	13.138	7.372	7.488	15.223
2	ProQ3D	QA339_2	13.569	7.638	7.873	15.384
3	BAKER-ROSETTASERVER	QA209_2	12.682	7.663	7.360	11.616
4	MULTICOM-CONSTRUCT	QA198_2	9.240	8.142	11.095	14.337
5	BAKER-experimental	QA403_2	13.192	8.268	7.659	12.008
6	MULTICOM-CLUSTER	QA075_2	8.886	8.307	10.647	14.456
7	QMEANDisCo	QA280_2	13.913	8.323	11.287	19.060
8	P3De	QA257_2	12.020	8.652	12.451	18.262
9	MUFOLD	QA081_2	12.557	8.691	9.579	16.809
10	VoroCNN-GEMME	QA406_2	15.682	8.701	8.124	18.223

## CONCLUSIONS

The ModFOLD8 server produces accurate estimates of local and global quality of 3D protein models, which are intuitively presented and freely accessible to all. Independent evaluations in the latest CASP experiments and the continuous CAMEO project have demonstrated that the ModFOLD server has maintained its position over the years as a leading resource for model quality estimation. With the advent of advanced deep learning methods, predicted tertiary structures of proteins are becoming increasingly accurate. However, it must be stated that even the very best 3D protein models from current publicly available servers often contain errors. In order to effectively utilise 3D models for further biological research, researchers must have confidence in their models and be able to identify any local regions that are likely to contain errors. Therefore, independent quality checking servers, such as ModFOLD8, are essential to build and maintain trust in 3D protein models and drive their wider adoption by life scientists.

## DATA AVAILABILITY

The ModFOLD8 server is freely available at: https://www.reading.ac.uk/bioinf/ModFOLD/.

## Supplementary Material

gkab321_Supplemental_File
