# Supplementary material for: ModFOLD8: accurate global and local quality estimates for 3D protein models
Source: Nucleic Acids Res. 2021 May 8;49(W1):W425–30. doi: 10.1093/nar/gkab321 (PMC8218196; doi:10.1093/nar/gkab321)
Supplement: gkab321_Supplemental_File [file gkab321_supplemental_file.pdf]

Supplementary Figure S1. ModFOLD8 neural network architecture and flow of data for local quality scoring. Scores for each residue in the model were fed into the input layer, taken from the 13 individual scoring methods using a sliding window of 5 residues (65 inputs, 33 hidden neurons, 1 output neuron). One variant of the network was trained to learn the S-score of the residue in the model and the other variant was trained to the lDDT score. The MLP (multilayer perceptron) function from RSNNS (https://cran.r-project.org/web/packages/RSNNS/) was used to build and train the network.

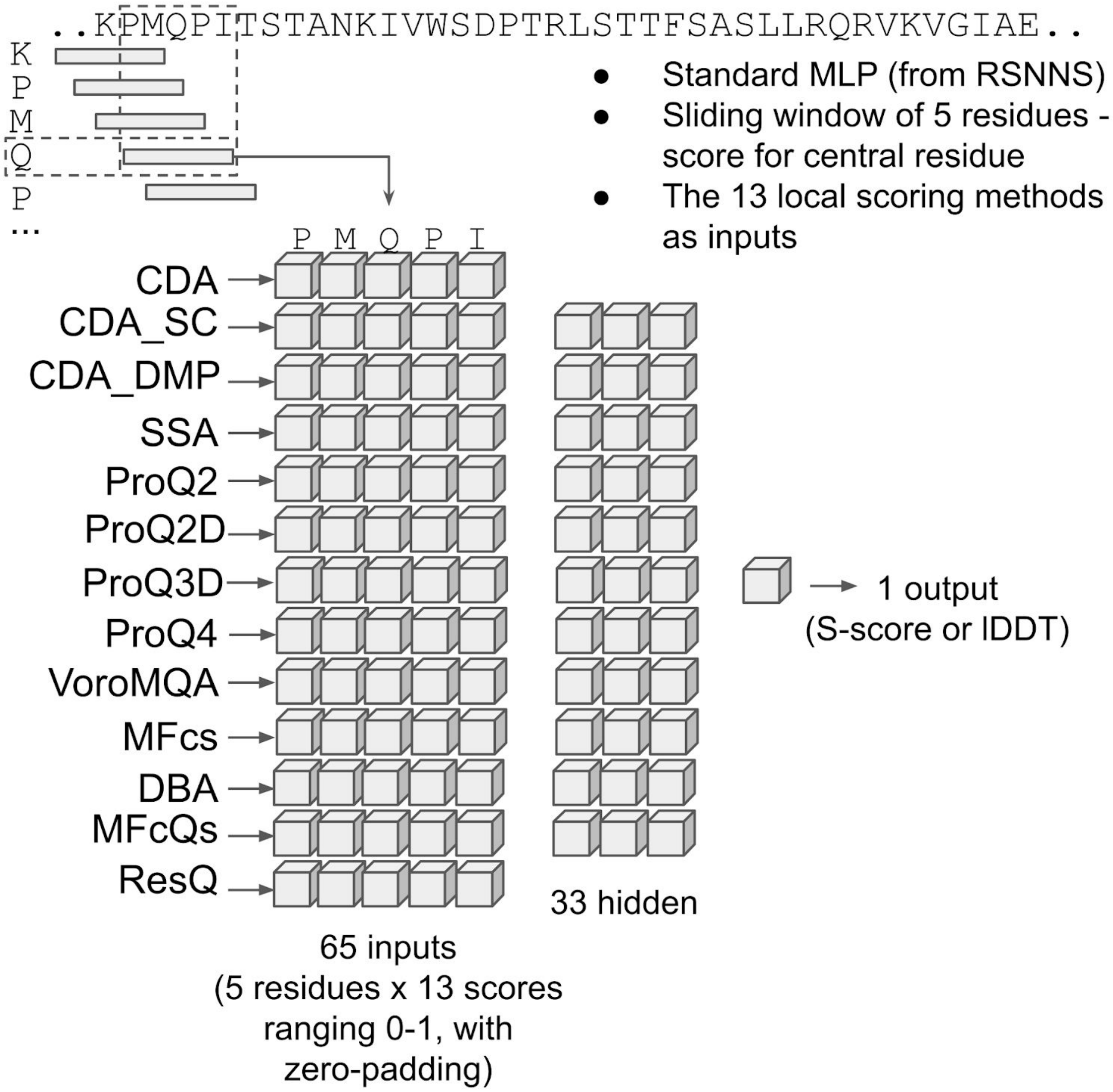

Supplementary Figure S2. Progressive increases in the accuracy of ModFOLD versions according to benchmarks on the CASP11 data set. **(A)** Increase in local model quality performance measured by Receiver Operating Characteristic (ROC) analysis Area Under the Curve (AUC), according to the lDDT score using a threshold of 0.6. **(B)** Increase in local model quality performance ROC analysis AUC evaluated using the S-score with a threshold of 3.5 Å. **(C)** Pearson correlation of predicted global scores and GDT_HA scores, showing the increasing accuracy of the ModFOLD_cor variants. **(D)** Cumulative GDT_HA scores of the top selected models, indicating the improvement of the ModFOLD_rank variants.

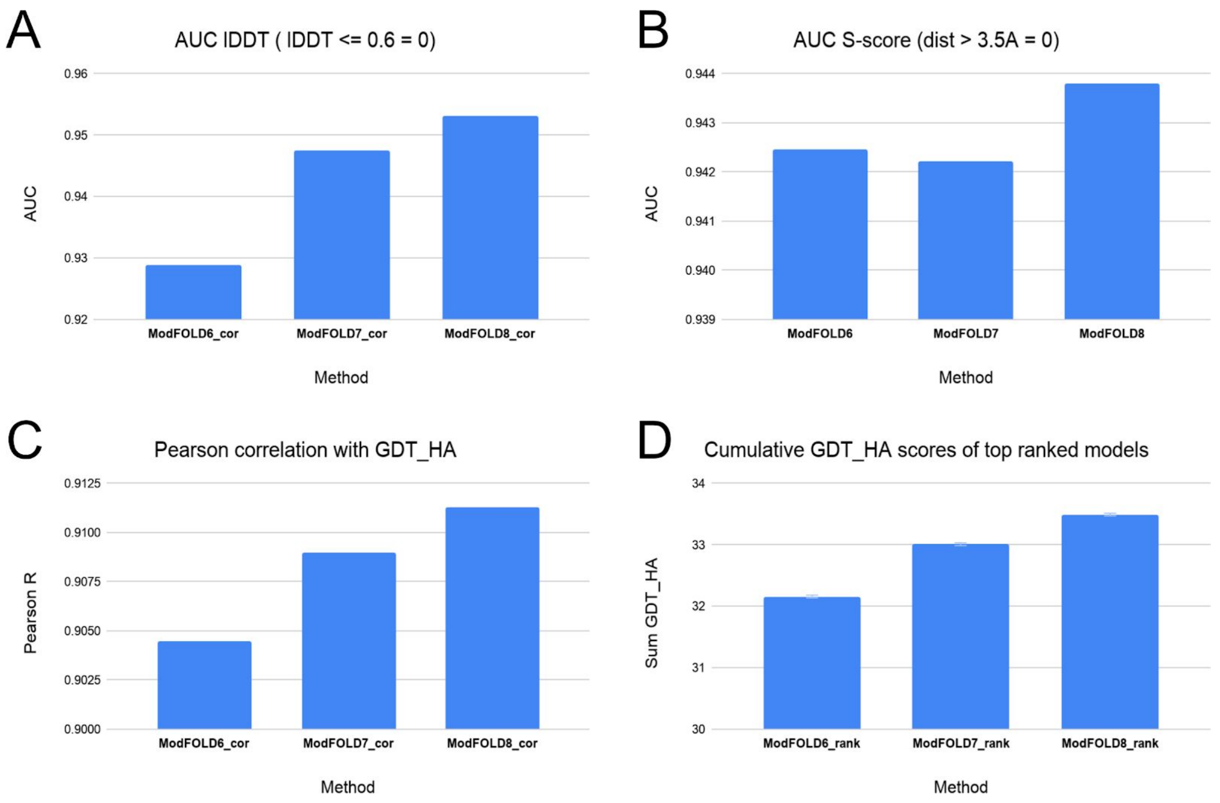

Supplementary Figure S3. Independent continuous benchmarking results showing the progressive increases in accuracy of the ModFOLD versions, from ModFOLD4 to ModFOLD8, according to CAMEO QE data. The area under the curve (AUC) of the Receiver Operating Characteristic (ROC) is calculated using an lDDT local score threshold of 60. ROC AUC*0,0.2, partial AUC of the ROC 'trimmed' at a FPR threshold of 0.2 and scaled between 0 and 1. The Precision vs Recall (PR) curve is also calculated using an lDDT local threshold of 60. PR AUC*0.8,1 is the partial AUC calculated considering Recall (or TPR) ranging between 0.8 to 1 and scaled between 0 and 1. **(A)** Performance plot for ModFOLD versions versus the best available public methods for 1-year of data [2020-01-17 - 2021-01-09] - "All" dataset (8426 models). **(B)** Performance plot for ModFOLD versions, 1-week of data [2021-01-16], "All" dataset (165 models). **(C)** Performance plot for ModFOLD versions, 1-month of data [2021-02-05 - 2021-02-27], "All" dataset (585 models). ModFOLD8 is listed as "Server 39", at the time of writing. Images of plots were downloaded from the CAMEO website: https://www.cameo3d.org/quality-estimation/.

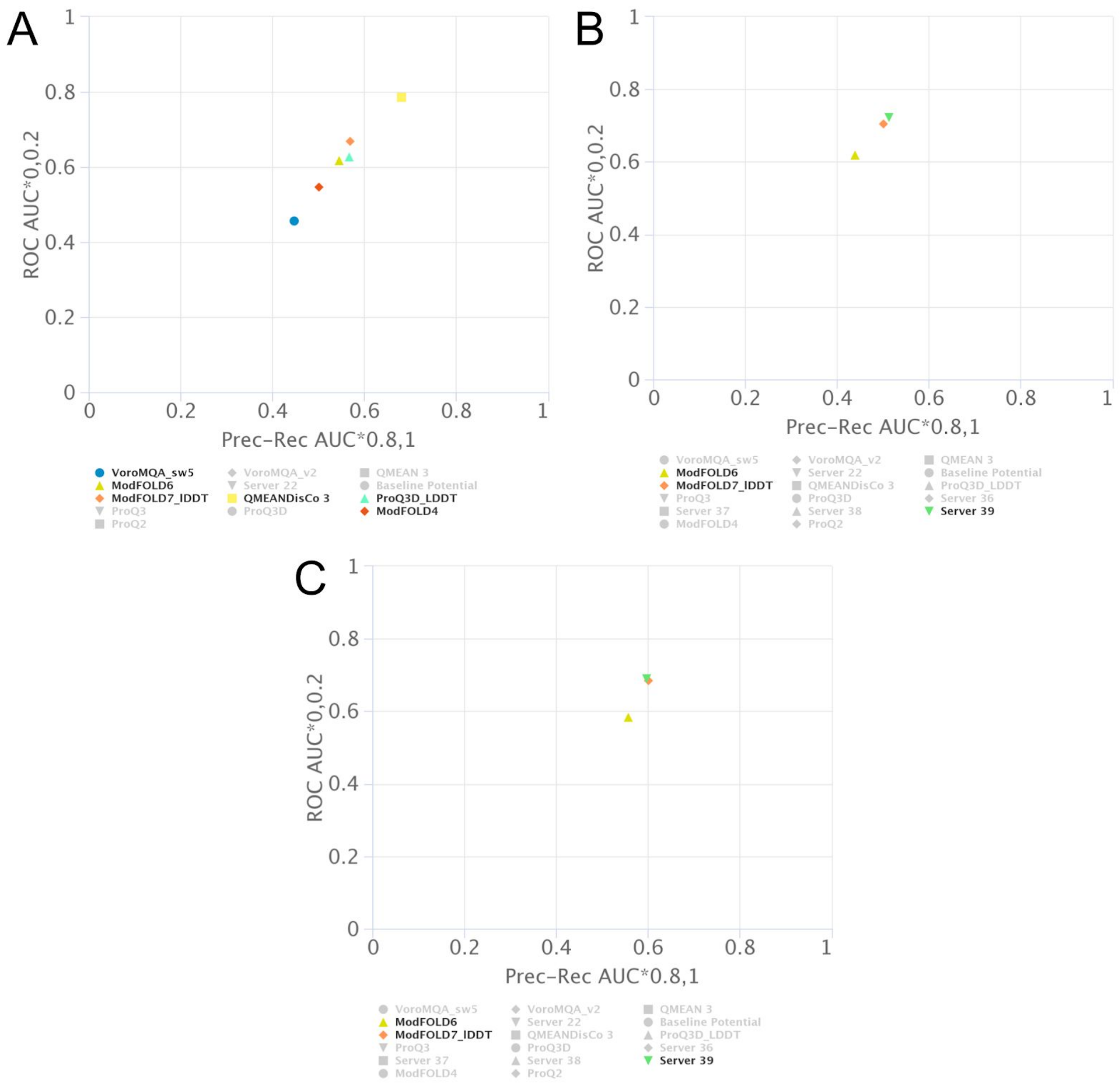

Supplementary Figure S4. ModFOLD8 local model quality results for the two SARS-Cov-2 targets from CASP14 and CASP Commons 2020 with structures in the PDB. Models in each panel are coloured according to the ModFOLD8 predicted local errors (left) and the observed distance from native (right). The temperature colouring scheme is used with colours from dark blue (indicating residues predicted to be close to the native structure <= 1Å) to red (indicating residues distant from the native structure >= 15Å). Images were rendered using PyMOL (http://www.pymol.org/). **(A)** AlphaFold2 model (427_1) for ORF8 (PDB ID 7jtl, CASP14 target T1064, CASP Commons target C1908). **(B)** AlphaFold model (156_1) for ORF3a domain 1 (PDB ID 6xdc, CASP Commons target C1905d1). **(C)** AlphaFold model (156_1) for ORF3a domain 2 (PDB ID 6xdc, CASP Commons target C1905d2).

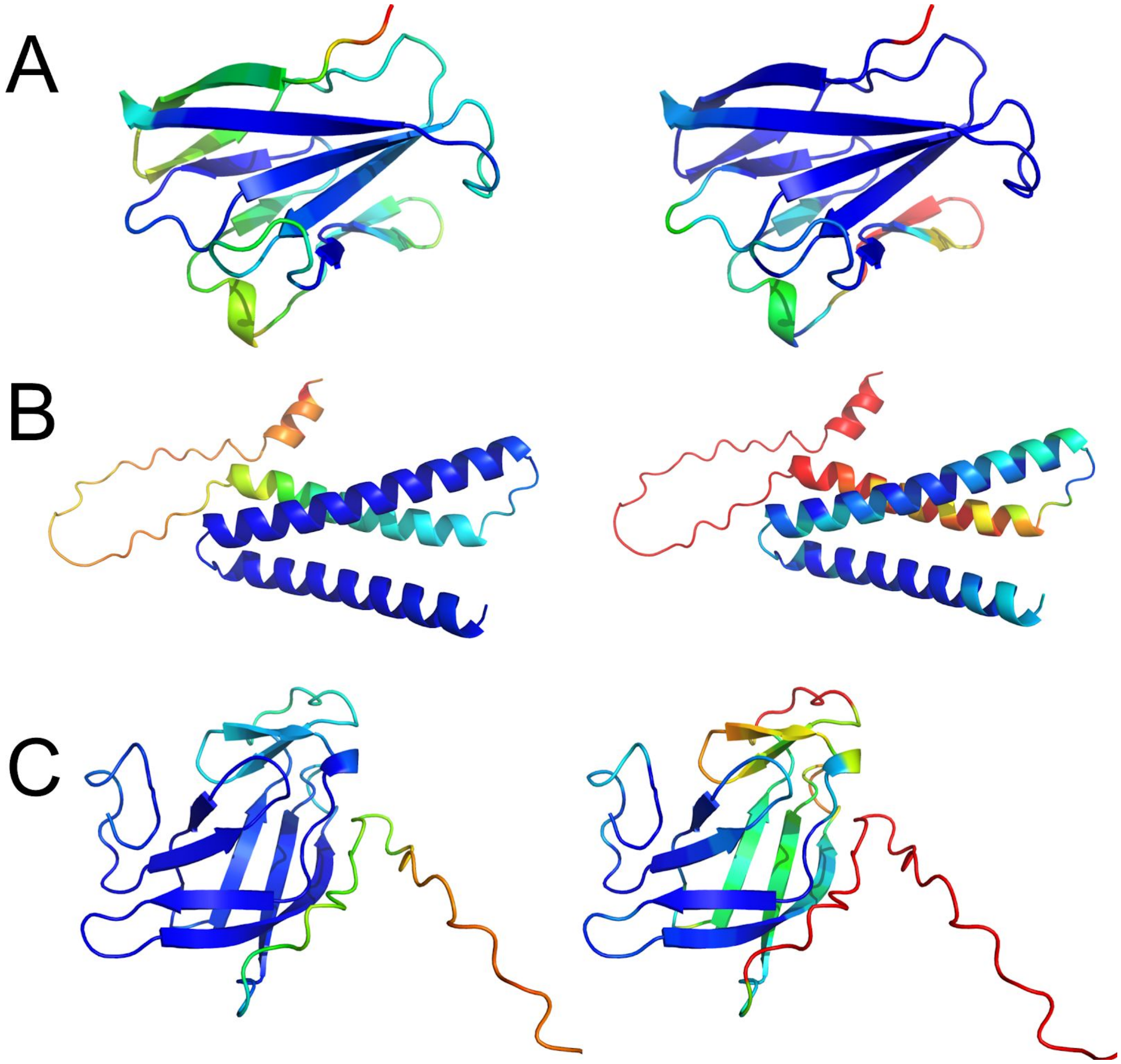

Supplementary Table S1. Independent continuous benchmarking results showing the progressive increases in accuracy of the ModFOLD versions according to CAMEO QE data. The area under the curve (AUC) of the Receiver Operating Characteristic (ROC) is calculated using an lDDT local score threshold of 60. ROC AUC0,1, pairs of True Positive Rate (TPR) and False Positive Rate (FPR) values, computed for all positive thresholds. ROC AUC*0,0.2, partial AUC of the ROC 'trimmed' at a FPR threshold of 0.2 and scaled between 0 and 1. The Precision vs Recall (PR) curve is also calculated using an lDDT local threshold of 60. PR AUC*0.8,1 is the partial AUC calculated considering Recall (or TPR) ranging between 0.8 to 1 and scaled between 0 and 1. Performance of ModFOLD versions versus the best available public methods for 1-year of data [2020-01-17 - 2021-01-09] - "All" dataset (8426 models). The table is sorted by the ROC AUC0,1 score. Data are from the CAMEO website (https://www.cameo3d.org/quality-estimation/).

**Predictor Name**	**ROC**		**PR**	
	**AUC0,1**	**AUC*0,0.2**	**AUC0,1**	**AUC*0.8,1**
QMEANDisCo 3	0.94	0.78	0.91	0.68
**ModFOLD7_lDDT**	0.9	0.66	0.83	0.57
ProQ3D_LDDT	0.89	0.62	0.82	0.57
**ModFOLD6**	0.88	0.61	0.81	0.55
**ModFOLD4**	0.85	0.54	0.76	0.5
VoroMQA_sw5	0.8	0.46	0.7	0.45

Supplementary Table S2. Independent continuous benchmarking results showing the progressive increases in accuracy of the ModFOLD versions according to CAMEO QE data. **(A)** Performance scores for ModFOLD versions, 1-week of data [2021-01-16], "All" dataset (165 models). **(B)** Performance scores for ModFOLD versions, 1-month of data [2021-02-05 - 2021-02-27], "All" dataset (585 models). ModFOLD8 is listed as “Server 39”, at the time of writing. The table is sorted by the ROC AUC0,1 score. Data are from the CAMEO website (https://www.cameo3d.org/quality-estimation/).

**A)** **Predictor Name**	**ROC**		**PR**	
	**AUC0,1**	**AUC*0,0.2**	**AUC0,1**	**AUC*0.8,1**
**ModFOLD8_lDDT (Server 39)**	0.91	0.72	0.85	0.51
**ModFOLD7_lDDT**	0.9	0.7	0.84	0.5
**ModFOLD6**	0.87	0.62	0.79	0.44

**B)** **Predictor Name**	**ROC**		**PR**	
	**AUC0,1**	**AUC*0,0.2**	**AUC0,1**	**AUC*0.8,1**
**ModFOLD8_lDDT (Server 39)**	0.9	0.69	0.86	0.6
**ModFOLD7_lDDT**	0.9	0.68	0.86	0.6
**ModFOLD6**	0.87	0.58	0.8	0.56

Supplementary Table S3. Official CASP14 local QA evaluation (Accuracy Self Estimates (ASE), stage 2 - best 150). The top 10 groups are shown. Table is sorted by the ASE score. Data are from: https://predictioncenter.org/casp14/qa2_ase.cgi

**Rank**	**Group**	**Model**	**ASE**
1	DAVIS-EMAconsensus	QA433_2	85.361
2	**ModFOLDclust2**	QA035_2	85.217
3	Yang_TBM	QA460_2	84.798
4	Yang-Server	QA140_2	84.234
5	UOSHAN	QA409_2	83.707
6	EMAP_CHAE	QA216_2	82.362
7	Wallner	QA379_2	81.042
8	**ModFOLD8_rank**	QA120_2	78.741
9	**ModFOLD8**	QA167_2	78.74
10	ProQ3D	QA339_2	76.779

Supplementary Table S4. Official CASP14 global QA evaluation (Difference from best, stage 2 - best 150). The top 20 groups are shown. Table is sorted by the GDT-TS score. Lower scores indicate higher performance. Data are from: https://predictioncenter.org/casp14/qa_diff2best.cgi

**Rank**	**Group**	**Model**	**GDT_TS**	**LDDT**	**CAD (AA)**	**SG**
1	MULTICOM-CONSTRUCT	QA198_2	7.356	5.436	2.923	7.006
2	MULTICOM-AI	QA275_2	7.924	6.748	3.489	8.07
3	MESHI	QA032_2	7.931	5.287	3.05	7.165
4	MULTICOM-CLUSTER	QA075_2	8.023	6.893	3.713	8.052
5	MUfoldQA_G	QA446_2	8.201	7.07	4.63	9.124
6	MESHI_consensus	QA214_2	8.404	6.147	3.459	7.513
7	BAKER-ROSETTASERVER	QA209_2	8.407	4.112	2.3	7.312
8	BAKER-experimental	QA403_2	8.453	4.885	2.48	7.987
9	**ModFOLD8**	QA167_2	8.497	6.985	3.848	9.087
10	Bhattacharya-Server	QA149_2	8.512	6.66	4.074	8.651
11	MULTICOM-HYBRID	QA187_2	8.606	6.686	3.651	8.312
12	Yang_TBM	QA460_2	8.795	7.108	4.103	8.476
13	Wallner	QA379_2	8.931	5.914	3.624	9.638
14	DAVIS-EMAconsensus	QA433_2	9.009	7.117	4.51	9.565
15	EMAP_CHAE	QA216_2	9.166	6.132	3.516	7.757
16	**ModFOLDclust2**	QA035_2	9.401	7.438	4.648	9.708
17	VoroCNN-GDT	QA153_2	9.69	4.708	2.407	8.002
18	GraphQA	QA210_2	9.83	8.543	4.47	10.067
19	Yang-Server	QA140_2	9.851	7.89	4.445	9.127
20	**ModFOLD8_rank**	QA120_2	10.238	8.057	4.39	9.689

Supplementary Table S5. Official CASP14 global QA evaluation (AUC stage 2 - best 150). The ability of methods to separate good models (accuracy score >=50) from bad (<50) according to GDT_TS, LDDT, CAD and SG scores is evaluated using the Areas Under the Curve (AUC). The top 15 groups are shown. Table is sorted by the LDDT AUC score. Data are from: https://predictioncenter.org/casp14/qa_aucmcc.cgi

Rank	Group	Model	GTD_TS AUC	LDDT AUC	CAD AUC	SG AUC
1	BAKER-ROSETTASERVER	QA209_2	0.85	0.912	0.919	0.921
2	BAKER-experimental	QA403_2	0.842	0.904	0.918	0.898
3	Bhattacharya-Server	QA149_2	0.909	0.897	0.923	0.924
4	MULTICOM-CONSTRUCT	QA198_2	0.918	0.889	0.882	0.893
5	3DCNN_prof	QA074_2	0.83	0.885	0.886	0.876
6	MULTICOM-CLUSTER	QA075_2	0.932	0.878	0.87	0.885
7	MULTICOM-HYBRID	QA187_2	0.914	0.876	0.881	0.893
8	MULTICOM-AI	QA275_2	0.912	0.875	0.876	0.883
9	**ModFOLD8_rank**	QA120_2	0.86	0.872	0.883	0.884
10	ProQ3D	QA339_2	0.825	0.87	0.886	0.875
11	P3De	QA257_2	0.859	0.869	0.866	0.878
12	Yang-Server	QA140_2	0.922	0.863	0.856	0.886
13	Yang_TBM	QA460_2	0.927	0.856	0.848	0.877
14	MULTICOM-DEEP	QA252_2	0.806	0.856	0.865	0.863
15	angleQA	QA391_2	0.801	0.856	0.84	0.847
16	Wallner	QA379_2	0.929	0.852	0.852	0.879
17	MUfoldQA_G	QA446_2	0.937	0.851	0.859	0.882
18	MESHI_consensus	QA214_2	0.94	0.849	0.861	0.878
19	EDN	QA203_2	0.772	0.844	0.902	0.866
20	MESHI	QA032_2	0.938	0.843	0.856	0.873

Supplementary Table S6. Official CASP13 local QA evaluation (Corr/MCC, stage 1 - select 20). The top 10 groups are shown. Table is sorted by the Corr. score. Score thresholds are shown in brackets. Data are from: https://predictioncenter.org/casp13/qa2_aucmcccorr.cgi

Rank	Group	Model	MCC(3.8)	MCC(5.0)	Corr.
1	**ModFOLD7**	QA275_1	0.589	0.612	0.599
2	**ModFOLD7_rank**	QA272_1	0.589	0.612	0.599
3	RaptorX-DeepQA	QA334_1	0.482	0.507	0.458
4	**ModFOLD7_cor**	QA213_1	0.449	0.506	0.434
5	Pcons	QA022_1	0.238	0.29	0.349
6	ProQ3D	QA139_1	0.352	0.339	0.335
7	UOSHAN	QA194_1	0.274	0.375	0.303
8	**ModFOLDclust2**	QA373_1	0.212	0.313	0.283
9	ProQ3	QA187_1	0.309	0.296	0.276

10	MASS2	QA415_1	0.226	0.198	0.257

Supplementary Table S7. Official CASP13 local QA evaluation (Corr/MCC/AUC, stage 2 - best 150). The top 10 groups are shown. Table is sorted by the AUC score. Score thresholds are shown in brackets. Data are from: https://predictioncenter.org/casp13/qa2_aucmcccorr.cgi

**Rank**	**Group**	**Model**	**MCC(3.8)**	**MCC(5.0)**	**Corr.**	**AUC(3.8)**
1	**ModFOLDclust2**	QA373_2	0.717	0.747	0.703	0.952
2	Davis-EMAconsensus	QA349_2	0.682	0.734	0.671	0.951
3	UOSHAN	QA194_2	0.727	0.748	0.713	0.945
4	**ModFOLD7_rank**	QA272_2	0.701	0.712	0.658	0.929
5	**ModFOLD7**	QA275_2	0.701	0.712	0.658	0.929
6	Pcomb	QA083_2	0.642	0.679	0.57	0.918
7	**ModFOLD7_cor**	QA213_2	0.645	0.643	0.594	0.904
8	Wallner	QA457_2	0.575	0.637	0.45	0.904
9	Pcons	QA022_2	0.606	0.648	0.488	0.903
10	RaptorX-DeepQA	QA334_2	0.592	0.626	0.582	0.88

Supplementary Table S8. Official CASP13 local QA evaluation (Accuracy Self Estimates (ASE), stage 1 - select 20). The top 10 groups are shown. Table is sorted by the ASE score. Data are from: https://predictioncenter.org/casp13/qa2_ase.cgi

**Rank**	**Group**	**Model**	**ASE**
1	RaptorX-DeepQA	QA334_1	87.957
2	UOSHAN	QA194_1	85.428
3	Davis-EMAconsensus	QA349_1	85.245
4	**ModFOLDclust2**	QA373_1	84.762
5	**ModFOLD7**	QA275_1	84.058
6	**ModFOLD7_rank**	QA272_1	84.056
7	Yang-Server	QA164_1	83.265
8	Pcons	QA022_1	82.71
9	Wallner	QA457_1	82.149
10	ProQ3D-TM	QA267_1	81.952

Supplementary Table S9. Official CASP13 local QA evaluation (ASE, stage 2 - best 150). The top 10 groups are shown. Table is sorted by the ASE score. Data are from: https://predictioncenter.org/casp13/qa2_ase.cgi

**Rank**	**Group**	**Model**	**ASE**
1	UOSHAN	QA194_2	87.755
2	**ModFOLDclust2**	QA373_2	86.588
3	Davis-EMAconsensus	QA349_2	86.01
4	RaptorX-DeepQA	QA334_2	84.407
5	**ModFOLD7**	QA275_2	83.357
6	**ModFOLD7_rank**	QA272_2	83.356
7	Pcons	QA022_2	82.766
8	Pcomb	QA083_2	80.499
9	Wallner	QA457_2	80.414
10	ProQ3D-TM	QA267_2	79.229

Supplementary Table S10. Official CASP13 global QA evaluation (Differences (predicted vs observed), stage 1 - select 20). The top 10 groups are shown. Table is sorted by the GDT_TS score. Lower scores indicate higher performance. Data are from: https://www.predictioncenter.org/casp13/qa_diff_mqas.cgi

Rank	Group	Model	GDT_TS	LDDT	CAD(AA)	SG
1	MUfoldQA_T	QA211_1	4.309	9.331	23.817	8.913
2	MUFoldQA_M	QA113_1	4.57	9.325	23.882	9.048
3	MUfoldQA_S2	QA107_1	4.848	7.682	21.363	8.538
4	UOSHAN	QA194_1	5.305	12.778	27.578	10.212
5	**ModFOLD7**	QA275_1	5.33	6.564	20.939	7.573
6	MULTICOM_CLUSTER	QA058_1	5.722	8.192	21.995	9.283
7	**ModFOLD7_cor**	QA213_1	5.795	5.832	19.894	7.937
8	Davis-EMAconsensus	QA349_1	6.14	13.469	28.319	10.668
9	RaptorX-DeepQA	QA334_1	6.223	13.48	27.918	10.056
10	MULTICOM-CONSTRUCT	QA243_1	7.287	5.798	18.936	9.255

Supplementary Table S11. Official CASP13 global QA evaluation (Differences (predicted vs observed), stage 2 - select 150). The top 10 groups are shown. Table is sorted by the GDT_TS score. Lower scores indicate higher performance. Data are from: https://www.predictioncenter.org/casp13/qa_diff_mqas.cgi

Rank	Group	Model	GDT_TS	LDDT	CAD(AA)	SG
1	UOSHAN	QA194_2	5.796	11.26	17.317	11.967
2	MUfoldQA_T	QA211_2	5.942	11.409	17.464	12.211
3	MUFoldQA_M	QA113_2	6.066	11.948	17.993	12.472
4	**ModFOLD7_cor**	QA213_2	7.14	9.944	15.361	12.763
5	**ModFOLD7**	QA275_2	7.291	10.063	15.495	12.617
6	Davis-EMAconsensus	QA349_2	7.327	12.164	18.541	14.198
7	MULTICOM_CLUSTER	QA058_2	7.654	8.157	12.273	12.466
8	**ModFOLDclust2**	QA373_2	7.669	12.1	18.221	14.435
9	MULTICOM-CONSTRUCT	QA243_2	8.507	7.098	10.715	12.258
10	MUfoldQA_S2	QA107_2	10.043	9.936	14.661	14.159

Supplementary Table S12. Official CASP13 global QA evaluation (Difference from the best, stage 1 - select 20). The top 10 groups are shown. Table is sorted by the GDT_TS score. Lower scores indicate higher performance. Data are from: https://www.predictioncenter.org/casp13/qa_diff2best.cgi

Rank	Group	Model	GDT_TS	LDDT	CAD(AA)	SG
1	**ModFOLD7_rank**	QA272_1	0.535	0.339	0.249	0.582
2	MUfoldQA_T	QA211_1	0.999	1.019	0.751	1.848
3	MUFoldQA_M	QA113_1	1.017	1.062	0.751	1.856
4	**ModFOLD7**	QA275_1	1.055	0.594	0.523	1.178
5	**ModFOLD7_cor**	QA213_1	1.055	0.746	0.566	1.582
6	MULTICOM_CLUSTER	QA058_1	1.108	0.968	0.693	1.573
7	MULTICOM-CONSTRUCT	QA243_1	1.606	1.198	0.871	2.067
8	RaptorX-DeepQA	QA334_1	1.808	1.793	1.146	2.827
9	FaeNNz	QA027_1	1.917	1.566	0.95	2.887
10	Pcomb	QA083_1	3.39	2.019	1.161	3.222

Supplementary Table S13. Official CASP13 global QA evaluation (Difference from the best, stage 2 - select 150). The top 10 groups are shown. Table is sorted by the GDT_TS score. Lower scores indicate higher performance. Data are from: https://www.predictioncenter.org/casp13/qa_diff2best.cgi

Rank	Group	Model	GDT_TS	LDDT	CAD(AA)	SG
1	MULTICOM_CLUSTER	QA058_2	5.162	3.804	3.188	5.898
2	UOSHAN	QA194_2	5.555	4.503	4.126	5.598
3	MUFoldQA_M	QA113_2	6.264	5.985	4.955	9.136
4	MULTICOM-CONSTRUCT	QA243_2	6.865	6.219	4.207	9.03
5	Davis-EMAconsensus	QA349_2	6.888	6.277	5.178	8.907
6	Bhattacharya-ClustQ	QA014_2	7.071	5.764	4.766	9.601
7	**ModFOLDclust2**	QA373_2	7.178	6.305	5.158	8.902
8	**ModFOLD7_rank**	QA272_2	7.525	4.343	3.22	8.02
9	MUfoldQA_T	QA211_2	7.536	5.741	4.781	8.761
10	RaptorX-DeepQA	QA334_2	7.595	6.477	5.224	8.857

Supplementary Table S14. Official CASP13 global QA evaluation (AUC, stage 1 - select 20). The top 10 groups are shown. The ability of methods to separate good models (accuracy score >=50) from bad (<50) according to GDT_TS, LDDT, CAD and SG scores is evaluated using the Areas Under the Curve (AUC). Table is sorted by the GDT_TS_AUC score. Data are from: https://www.predictioncenter.org/casp13/qa_aucmcc.cgi

Rank	Group	Model	GDT_TS AUC	LDDT AUC	CAD AUC	SG AUC
1	**ModFOLD7_cor**	QA213_1	0.977	0.937	0.866	0.939
2	**ModFOLD7**	QA275_1	0.977	0.934	0.855	0.932
3	MULTICOM-CONSTRUCT	QA243_1	0.969	0.942	0.868	0.943
4	LamoureuxLab	QA067_1	0.968	0.912	0.873	0.926
5	**ModFOLD7_rank**	QA272_1	0.967	0.919	0.881	0.917
6	MUfoldQA_S2	QA107_1	0.957	0.941	0.847	0.94
7	RaptorX-DeepQA	QA334_1	0.956	0.92	0.838	0.932
8	FaeNNz	QA027_1	0.952	0.971	0.951	0.969
9	MESHI	QA197_1	0.941	0.905	0.893	0.924
10	MUfoldQA_T	QA211_1	0.941	0.897	0.822	0.906

Supplementary Table S15. Official CASP13 global QA evaluation (AUC, stage 2 - select 150). The top 15 groups are shown. The ability of methods to separate good models (accuracy score >=50) from bad (<50) according to GDT_TS, LDDT, CAD and SG scores is evaluated using the Areas Under the Curve (AUC). Table is sorted by the GDT_TS_AUC score. Data are from: https://www.predictioncenter.org/casp13/qa_aucmcc.cgi

Rank	Group	Model	GDT_TS AUC	LDDT AUC	CAD AUC	SG AUC
1	MUfoldQA_T	QA211_2	0.961	0.929	0.909	-
2	MUFoldQA_M	QA113_2	0.959	0.922	0.9	-
3	UOSHAN	QA194_2	0.959	0.917	0.898	0.928
4	Davis-EMAconsensus	QA349_2	0.954	0.909	0.89	-
5	Pcomb	QA083_2	0.953	0.912	0.899	-
6	Pcons	QA022_2	0.95	0.891	0.87	-
7	**ModFOLDclust2**	QA373_2	0.947	0.904	0.886	-

8	RaptorX-DeepQA	QA334_2	0.946	0.883	0.868	-
9	Wallner	QA457_2	0.945	0.896	0.885	0.906
10	MULTICOM-CONSTRUCT	QA243_2	0.945	0.931	0.914	-
11	**ModFOLD7_cor**	QA213_2	0.942	0.907	0.886	-
12	Bhattacharya-ClustQ	QA014_2	0.942	0.949	0.932	-
13	**ModFOLD7**	QA275_2	0.941	0.908	0.891	-
14	MULTICOM_CLUSTER	QA058_2	0.937	0.922	0.904	-
15	**ModFOLD7_rank**	QA272_2	0.93	0.91	0.906	-
